# Complexities of Dengue Fever: Pathogenesis, Clinical Features and Management Strategies

**DOI:** 10.15190/d.2024.8

**Published:** 2024-06-30

**Authors:** Maheen Nasir, Javeria Irfan, Aimen Binte Asif, Qudsia Umaira Khan, Haleema Anwar

**Affiliations:** CMH Lahore Medical and Dental College, Pakistan

**Keywords:** Dengue, dengue fever, dengue virus, dengue virus receptor, dengue vaccine, dengue hemorrhagic fever.

## Abstract

Dengue fever, transmitted through the bite of infected Aedes mosquitos, poses a significant global threat, particularly in the tropical and subtropical region. In this review, we aim to summarize the existent literature on dengue virus infection and to enlighten the reader on recent advances and knowledge. Dengue virus infection can cause a spectrum of clinical manifestations, ranging from asymptomatic or mild illness to more severe and potentially life-threatening complications. Pathogenesis of dengue is based on viral and host factors. Viral factors include NS1 antigen and genomic factors. Host factors include antibody dependent enhancement, anti-NS1 antibodies, cytokines, cross reactive T-Cell response, HLA allele variation and non-HLA mediated polymorphisms. The clinical picture of dengue is described on the basis of WHO 1997 and 2009 criteria. It is classified into dengue fever, dengue hemorrhagic fever (DHF), and dengue shock syndrome (DSS). Life-threatening complications can develop in severe cases, and this includes renal complications such as acute kidney injury (AKI) and hepatic complications such as hepatic dysfunction and in rare cases, fulminant hepatic failure. Neurological complications, cardiac complications and respiratory distress syndrome have also been reported. Treatment methods include targeting the dengue vector and Carica papaya, a natural remedy with antiviral properties. Additionally, the role of corticosteroids, intravenous immunoglobulins, and mast cell inhibitors has been explored in dengue treatment, aiming to reduce severity. Novel approaches involve drugs targeting dengue proteins and host factors necessary for the virus's life cycle, offering potential avenues for more targeted therapeutic interventions. In recent years, significant progress has been made in the development of vaccines against dengue, with Sanofi Pasteur’s Dengvaxia being the first licensed vaccine approved for use. Utilizing various approaches such as recombinant proteins, viral vectors and viral like particles, various alternatives have been provided which aim to be safer substitutes to Dengvaxia while maintaining the effectiveness. A review on dengue is essential for clinicians and healthcare professionals to stay updated on diagnostics, treatment protocols and prevention strategies.

## SUMMARY

1. Introduction

2. Pathogenesis

3. Clinical Findings

4. Complications of Dengue Fever

5. Treatment

6. Dengue Vaccine

7. Conclusion

## 1. Introduction

Dengue virus (DENV) is a single-stranded RNA virus of the Flaviviridae family and the Flavivirus genus^1,2^. The distinctive feature of arboviruses, or arthropod-borne viruses, is their ability to spread from arthropod vectors to vertebrate hosts despite their taxonomically different nature. They are categorized based on replicative methods, morphology, and antigenic connections.

Arboviruses belong to the Togaviridae, Flaviviridae, Bunyaviridae, Rhabdoviridae, Orthomyxoviridae, and Reoviridae virus families^3-6^. This virus is spread through the bite of an Aedes species female mosquito, primarily Aedes aegypti but also infrequently Aedes albopictus^3^. Four dengue virus serotypes (DEN-1, DEN-2, DEN-3, and DEN-4) belonging to the Flavivirus genus cause dengue infection in humans.

**Figure 1:**
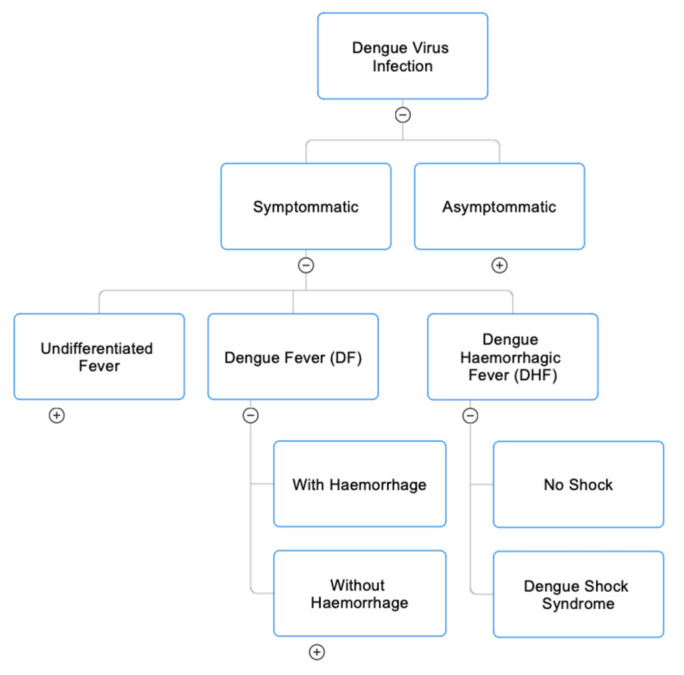
Spectrum of clinical manifestations seen with dengue virus infection^5-7^

Dengue fever (DF), dengue hemorrhagic fever (DHF), and dengue shock syndrome (DSS) are the three categories into which symptomatic dengue virus infection has been divided, according to the WHO classification from 1997 as shown in Figure 1. Dengue without warning signs, dengue with warning signs (abdominal pain, persistent vomiting, fluid accumulation, mucosal bleeding, lethargy, liver enlargement, increasing hematocrit with decreasing platelets), and severe dengue are the three categories of dengue patients according to the revised WHO classification of 2009^4^. The symptoms of DF, an acute febrile illness, include headaches, leukopenia, rash, and pains in the muscles, joints, and bones. The four main clinical signs of DHF are high fever, bleeding, frequently accompanied by hepatomegaly, and, in extreme situations, circulatory collapse. A significant amount of plasma leakage may cause hypovolemic shock in some of the affected people^5^. Whereas in DSS, there is a risk of serious bleeding, shock, and up to 20% fatality if treatment is not received^6^.

Dengue fever, a mosquito-borne infectious disease prevalent in tropical and subtropical regions, affects an estimated 3.6 billion individuals globally. The dengue virus (DENV) causes a substantial burden, with up to 390 million infections and 96 million symptomatic cases each year. The first reports of dengue or dengue-like disease date back to 1780 in Madras, India; however, the first virologically confirmed dengue fever epidemic in India took place in Calcutta and along the Eastern Coast of India in 1963–1964. The Philippines was the first place where DHF, a severe illness derived from DF patients, was initially documented in 1953. Eight DHF was theorized to be the result of numerous DENV infections because patients in the Philippines in 1956 had different serotypes (DENV-2, 3 and 4) isolated from them. During an epidemic in Bangkok, Thailand in 1958, people with multiple DENV infections were also isolated^8,9^. The incubation period ranges from three to fourteen days, with an average duration of five to seven days, during which viremic hosts can transmit the virus to mosquitoes for five to twelve days. Notably, asymptomatic cases, although capable of spreading the virus, often go undetected in global surveillance systems.

Dengue is endemic in approximately 125 countries, with transmission reported across all World Health Organization (WHO) regions. International travel facilitates the importation of cases into both endemic and non-endemic countries. In non-tropical areas, Aedes albopictus serves as the primary vector, with a broader host range, while Aedes aegypti predominates in tropical regions, posing a risk to travelers through mosquito bites^7^ .Frequent outbreaks and a high prevalence of dengue disease put a severe strain on health services and the nation’s economy. The three primary methods for preventing and controlling the spread of the dengue virus are vector control, case management, and case detection. There is currently a dengue vaccine on the market, and more vaccines are being developed. To properly use current and new preventive and control techniques, judgments on the burden, prevalence, incidence, and geographic distribution of dengue disease must be made. Considering this, we estimated the illness burden of dengue fever by a systematic study^8^.

## 2. Pathogenesis

Dengue fever is a mosquito-borne disease caused by one of the four antigenically distinguishable serotypes i.e. DENV-1 to DENV-4. Pathogenesis is mediated by viral and host factors as shown in Table 1. Viral factors include NS1 antigen, subgenomic flavivirus RNA and other genomic factors while host factors include cross-reactive T-Cell response, antibody-dependent enhancement, anti-NS1 antibodies, cytokines, HLA and non-HLA mediated polymorphisms.

**Table 1:** Viral and host factors mediating pathogenesis of dengue fever^10-20^

Host Factors	Viral Factors
Antibody dependent enhancement	NS1 antigen
Cross reactive T cell response	sfRNA
Anti-NS1 antibodies	Genotypic variation
HLA alleles	
Non HLA polymorphisms Cytokines	

### 2.1 Cross-Reactive T Cell Response

On infection with DENV, the ability of dendritic cells to mature and migrate will be interrupted by NS1 protein through regulation of associated gene expression^10^. Dendritic cells then express the target antigen to CD8+ AND CD4+ T-cells. CD4+ cells act on structural and NS1 proteins thus assisting in humoral response, T-cell mediated memory response and producing cytotoxic effect. Whereas CD8+ cells act on non-structural proteins and produce cytolytic effects on infected cells directly.

During a secondary DENV infection, CD8+ T-Cells with high affinity for the infecting virus are selectively activated and produce large numbers of inflammatory cytokines like IFN-γ, IL-13 and TNF-α. Those with low affinity for the heterologous infecting virus, however, are favorably multiplied. They lose their cytotoxic ability so viral clearance is prolonged but produces large numbers of inflammatory cytokines which play a major role in increased vascular permeability^11^.

Moreover, the heterologous DENV infection activates memory T-cells selective for the primary DENV strain rather than I T-cells. This phenomenon is called original antigenic sin^12^.

### 2.2 Antibody-Dependent Enhancement (Ade)

Infection with one DENV serotype provides lifetime protection against it while only short-term cross-reactive protection from other serotypes. When a secondary infection occurs, sub or non-neutralizing antibodies bind to DENV and ease its entrance into host cells by phagocytosis thus enhancing the virulence of the heterotypic strain. ADE occurs in infants as well due to the interaction between maternal antibodies and primary infection. ADE causes a high risk of severe infection in those who were infected primarily because of the production of low levels of sub-neutralizing antibodies against other serotypes^13^.

It has two mechanisms:

- Intrinsic ADE acts on the innate immune system to down-regulate levels of type-1 interferon, interleukin-12, interferon-γ and TNF thereby reducing the antiviral capability of DENV. This increases the burst size i.e. viral release from host cells.
- Extrinsic ADE refers to the increased number of infected cells when antibodies fall below their neutralizing ability.

### 2.3 Soluble Non-Structural Protein 1 Antigen (Sns1)

sNS1 protein is a glycoprotein existing as a dimer on the surface of DENV and secreted as a hexamer from infected cells. It can be detected in the bloodstream acute dengue so is used as a diagnostic marker^16^. It plays a part in viral morphogenesis and replication. Its role in the pathogenesis of dengue includes:

- sNS1 binds to endothelium and increases production of heparanidase and sialidase that disturb its normal structure. It also breaks the intracellular junctions by clathrin-mediated internalization or phosphorylation. This causes vessel damage and plasma leakage.
- Direct binding to TLR-4 increases expression of various cytokines and vasoactive amines e.g. interleukin 6 or TNF- alpha which increase the risk of vascular disorders leading to severe dengue.
- It causes fixation of many complement system components like membrane attack complex (MAC) and mannose-binding lectin, decreasing its ability to neutralize viruses and stopping activation by the lectin pathway.

### 2.4 Anti-Ns1 Antibodies

The presence of these antibodies causes immune activation and release of many inflammatory mediators like interleukin-6, interleukin-8 and MCP-1. They produce effects that may lead to DHF^19^. In severe dengue, hepatic damage is seen due to anti-NS1 antibodies as demonstrated in mice, the liver of whom showed the antibodies deposited in vascular endothelium and macrophages when immunized passively. When actively immunized the mice's liver showed hepatic fibrosis, and fatty liver and liver enzymes were raised^20^.

### 2.5 Genomic Factors

Certain DENV strains produce a greater risk of epidemics due to increased replicative potential in humans or mosquitoes. Further, the sequence of infection may affect the pathogenic ability of a specific serotype^21^. For example, DENV-1 results in higher viremia in contrast to DENV-2 and DENV-3. The Southeast Asian genotype of DENV-2 is more virulent and replicates at a higher titer as compared to the indigenous American DENV-2 genotype and leads to a more severe disease. Higher levels of dengue virus RNA are observed in in DHF patients. Moreover, subgenomic flavivirus RNA (sfRNA) can inhibit interferon-related translation^19^.

Human Leukocyte Antigen type 1 (HLA-1) causes increased severity of dengue while HLA class type 2 (HLA-2) and HLA-DR4 are protective against DHF. Factors not related to HLA include Fcγ receptor II (FcγRII, CD32), Vitamin D receptor (VDR) and Human Platelet Antigen (HPA). Other genetic polymorphisms such as the tumor necrosis factor (TNF)-308A allele increased the risk of severe dengue whereas the tumor necrosis factor (TNF)- 238 allele was protective^21^.

### 2.6 Cytokines

The excessive production of cytokines like TNF-alpha and interferon-γ from memory T cells during secondary infection activate polymorphonuclear monocytes. These then produce more inflammatory cytokines that increase vascular permeability^22^. Cytokines including CCL2, CCL5, CCL20 and CXCL1 are found to be raised in dengue. They disrupt lipid metabolism and alter membrane permeability. HMGB-1 and ICAM-1 were found to be correlated to the severity of the disease. HMGB-1 increases amounts of other inflammatory cytokines while ICAM-1 is an intercellular adhesion molecule^23^.

## 3. Clinical Findings

### 3.1 Classification of Dengue

According to 1997 WHO guidelines, the classification of dengue is divided into^24^: · Dengue fever (DF): Fever associated with two or more of the given features: headache, myalgia, retro-orbital pain, arthralgia, rash, leucopenia or hemorrhagic indications. · Dengue hemorrhagic fever (DHF): Hemorrhages, low platelet counts and plasma leakage. · Dengue shock syndrome (DSS): Hemorrhages, low platelet count, plasma leakage along with circulatory failure i.e. tachycardia, hypotension, pedal oedema, shortness of breath. The WHO guidelines in 2009 revised the criteria and divided dengue into non-severe and severe forms^25^. Non-severe dengue is further divided into those without warning signs and those with warning signs. Manifestations of non-severe dengue without warning signs include fever with nausea and vomiting, rash, body pain, positive tourniquet test or leucopenia. Warning signs included abdominal pain, mucosal bleeding, vomiting, hepatomegaly and findings of fluid accumulation. Severe dengue was presented as hepatomegaly, abdominal pain, bleeding, pleural effusion, ascites, increased hematocrit, high AST levels and decreased platelet count. This implied impending plasma leakage leading to decreased tissue perfusion and shock^26^.

### 3.2 Clinical Course

The clinical course of the disease follows three stages^27^: · Febrile phase: It lasts 2 to 7 days. It presents with sudden high fever, headache, arthralgia, myalgia, facial flushing, blanching macular skin rash, sore throat, nausea, vomiting, elevated liver enzymes and anorexia^28^. The temperature pattern is biphasic. · Critical phase. It does not occur in patients with DF but in those with DHF. It is marked by increased capillary permeability with increased hematocrit. Decreasing platelet count and progressive leukopenia occur before significant plasma loss. Significant plasma leakage leads to hypovolemic shock which presents with warning signs like subnormal temperature, sweaty hands and rapid thread-like pulse. Extended shock causes organ dysfunction, metabolic acidosis and disseminated intravascular coagulation thus, leading to severe dengue^29^. · Recovery phase: Lasts for 48 to 72 hours. It is characterized by reabsorption of extravascular fluid back into the vessels. The appetite is improved, gastrointestinal symptoms are relieved, and bradycardia, diuresis, pruritus and a generalized rash are seen (islands of white in a sea of red)^30^. Expanded dengue syndrome occurs when multiple organs are involved other than the plasma leakage. They do not fit into DHF or DSS. Organ systems involved include cardiovascular, kidneys, hematological, and gastrointestinal. Other than these, rhabdomyolysis and upper limb compartment syndrome have also been reported in several cases^31^.

## 4.Complications of Dengue Fever

Dengue fever, caused by the dengue virus and transmitted by Aedes mosquitoes, generally causes a mild, self-limiting illness which resolves on its own with supportive care. However, in some cases, especially in areas where it is endemic, the illness can rapidly progress to severe dengue which can be recognised by persistent vomiting, abdominal pain and bleeding under the skin (Table 2).

### 4.1 Neurological complications

Neurological complications of dengue, although incredibly rare, can occur in severe cases and are increasingly being recognized. Neurological syndromes of dengue, a neurotropic virus, occur due to direct invasion of the virus or due to the systemic effects caused by the infection^32^.

Most common neurological complications of dengue are encephalopathy and encephalitis^33^. Encephalopathy occurs in severe cases of dengue and signifies generalized brain dysfunction. It presents with altered levels of consciousness, seizures, confusion and irritability and may be associated with metabolic disturbances in the body, hypoxia or direct viral involvement. Similarly, encephalitis occurs due to direct invasion of the virus into the brain tissue which causes inflammation of the brain, resulting in symptoms such as seizures, headaches, and focal neurologic deficits. Among the rare neurologic complications of dengue is acute transverse myelitis (ATM) characterized by inflammation of the spinal cord and typically presents with sensory deficits, motor weakness and dysfunction of the bladder and the bowel^34^. Sudden neurological deficits including weakness in one half of the body, slurred speech and loss of consciousness can occur due to cerebral hemorrhage, the risk of which is increased in dengue due to impaired blood clotting^34^. Guillain Barre, an autoimmune disease can occur following the viral infection and it presents with ascending paralysis, starting from the legs and progressively moving upwards^35^. In some cases, dengue fever also has long-term neurological sequelae leading to cognitive impairment, memory deficits and altered behavior.

### 4.2 Gastrointestinal complications

Gastrointestinal complications of dengue include gastrointestinal bleeding, intestinal perforation, acute pancreatitis, hepatitis and in severe cases, fulminant hepatic failure. Among the early signs of dengue fever are nausea and vomiting which may persist throughout illness and may cause dehydration if fluids are not adequately replenished. Abdominal pain in dengue fever may be due to inflammation of the gastrointestinal system, liver failure or collection of fluid in the abdomen. Gastrointestinal bleeding is a manifestation of dengue hemorrhagic fever or dengue shock syndrome and indicates severe progression of the disease^36^. It may present with hematemesis or melena, blood in vomit or black tarry stools, respectively. If severe, excessive gastrointestinal bleeding can cause intestinal perforation which is a surgical emergency and requires urgent exploratory surgery to prevent the development of peritonitis and septic shock. Involvement of the pancreas and gallbladder is rare in dengue infection but it may occur in severe cases, manifested as pancreatitis and acalculous cholecystitis, respectively^37,38.^. Hepatic involvement in dengue is signified by elevated liver enzymes, jaundice, enlarged liver and accumulation of fluid in the abdominal cavity. Hepatic dysfunction occurs due to direct viral damage to the hepatic cells or secondary effects of response to the virus. Hepatic involvement due to dengue infection is characterized by higher levels of AST as compared to ALT due to the release of AST from hepatocytes damaged by the dengue infection^39^. Liver damage was most associated with serotypes DENV-3 and DENV-4 and was more common in women^40^.

### 4.3 Cardiac complications

Cardiac complications of dengue fever are rare and include myocarditis, arrhythmias, pericardial effusion, acute coronary syndrome (ACS), hypotension and shock. Arrhythmias associated with DHF have been reported and are due to the disruption of the normal electrical activity of the heart. Arrhythmias associated with dengue fever include atrioventricular block, atrial fibrillation, tachycardia and bradycardia, which manifest as irregular heartbeats^41^. Arrhythmias generally resolve with the infection but can further increase the risk of complications such as stroke or heart failure. Another commonly reported cardiac complication of dengue fever is myocarditis which is due to inflammation of the heart muscle and presents with shortness of breath, chest pain and fatigue^42^. Pericardial involvement in dengue fever involves pericardial effusion and pericarditis which are due to inflammation of the pericardium, the surrounding outer covering of the heart. Pericarditis is characterized by sharp stabbing pain in the chest and pericardial effusion may present with signs of cardiac tamponade if the amount of fluid around the heart is significant. In severe cases, individuals may suffer from long-term cardiac complications which include persistent arrhythmias and chronic dysfunction of the heart muscle known as cardiomyopathy.

### 4.4 Respiratory complications

In severe cases, the progression of dengue fever can lead to the development of acute respiratory distress syndrome (ARDS). ARDS is a life-threatening condition and is due to severe inflammation of the lung and concomitant impaired oxygenation^41^. ARDS is an emergency and can lead to respiratory failure, requiring ventilatory support. Severe dengue can lead to increased vascular permeability leading to extravasation of fluid (pulmonary edema) which impairs gas exchange and causes difficulty breathing and distress. The respiratory distress in dengue may be increased due to exacerbation of asthma, development of viral or bacterial pneumonia because of the impaired immune system, and accumulation of fluid in pleural space leading to pleural effusion. In a few cases, particularly in the pediatric population, dengue fever can cause inflammation of small airways leading to the development of bronchiolitis which presents with wheezing and signs of respiratory distress.

### 4.5 Hematological complications

The most prominent features of dengue fever, particularly in severe cases are the hematological complications. The hallmark hematological complication of dengue is thrombocytopenia, and decreased platelet count, which is due to both decreased production and increased destruction of platelets. Thrombocytopenia leads to bleeding tendencies and predisposes a patient to bleed of various types, such as petechiae, ecchymosis and more severe life-threatening manifestations of hemorrhage. In severe cases of dengue, due to endothelial injury which affects hemostasis, there is concomitant activation of coagulation due to the release of cytokines and fibrinolysis due to the formation of fibrin. A relative imbalance in the coagulation system can lead to disseminated intravascular coagulation which can cause widespread thrombosis and bleeding. Microvascular thrombosis can cause multi-organ ischemia and eventually lead to failure^43,44^. Dengue fever can cause suppression of the bone marrow leading to exacerbation of thrombocytopenia, development of anemia and dysfunction of the immune system. Another hematological complication is the development of hypovolemic shock the development of which is contributed by the leakage of plasma leading to hemoconcentration.

### 4.6 Renal complications

Hypovolemia due to plasma leakage, decreased blood supply to the kidneys and direct viral effect can lead to impairment of the kidney function and development of acute kidney injury (AKI)^45^. AKI leads to the retention of waste products and fluid imbalance in the body. Damage to the kidney can affect the filtration barrier and lead to proteinuria and severe bleeding and thrombocytopenia can lead to hematuria. Formation of small blood clots, as seen in severe cases of dengue, known as thrombotic microangiopathy can lead to impairment of kidney function and AKI. Electrolyte imbalances such as hyponatremia, hyperkalemia and acid-base abnormalities can further deteriorate kidney function and lead to renal complications. In cases of recurrent severe dengue or those with AKI, there is a risk of long-term kidney damage and the eventual development of chronic kidney disease.

## 5. Treatment

### 5.1 Fluid Management

Patients can be divided into four distinct categories (adapted from WHO, 2012).

- A normal heart rate and blood pressure of more than 20 mmHg.
- A pulse pressure or hypotension of less than 20 mmHg.
- Shock.
- Shock despite crystalloid solution fluid resuscitation.

The main form of therapy for groups 1-3 is crystalloid solution, which has certain limitations due to its increased capacity to leak into the third space, which may result in pulmonary edema, ascites, or pleural effusion.

According to a study conducted on Vietnamese children (Wills et al., 2005), severe shock should be treated with starch rather than dextran, whereas moderately severe shock should be treated with fluid therapy using Ringer’s lactate solution^46^. Colloids have the potential to expand volume in addition to the actual fluid volume infused. Colloid molecules encourage fluid retention in the intravascular compartment and raise plasma oncotic pressure. The average molecular weight of the colloid molecule controls the effect’s size, while circulation retention time controls how long the effect lasts. The plasma-volume-expanding capacity of crystalloids is correlated with sodium content. In principle, Ringer’s lactate (131 mM) may not perform as well as NaCl 0.9% (154 mM)^47,48^. Furthermore, there is a chance that infusing significant amounts of Ringer’s lactate will exacerbate tissue acidosis and lactate buildup. When treating children with DSS, Wills et al. tested Ringer’s lactate with two colloids and found no discernible benefit of the colloid over lactate infusion^49^.

**Table 2:** Complications of Dengue Fever^32-45^

Neurological	Cardiac	Renal	Respiratory	GI and Hepatic	Hematological	Long Term
Encephalopathy	Myocarditis	Proteinuria	Pulmonary edema	Gastrointestinal bleeding	Thrombocytopenia	Post-dengue fatigue syndrome
Encephalitis	Pericarditis	Hematuria	Pleural effusion	Intestinal perforation	Disseminated intravascular coagulation	Chronic Kidney disease
Seizures	Arrhythmia	Acute Kidney Injury	Acute respiratory distress syndrome (ARDS)	Acute pancreatitis	Bone marrow Suppression	Cognitive impairment
Guillain-Barré syndrome	Pericardial effusion	Hyperkalemia	Bronchiolitis	Jaundice	Anemia	Joint pain
Transverse myelitis	Acute coronary syndrome			Hepatits		
	Hypotension			Fulminant hepatic failure		
	Shock					

### 5.2 Drugs targeting dengue proteins

#### 5.2.1 Entry fusion inhibitors

The E protein, which binds to a variety of cellular receptors, is the main method by which the Dengue Virus (DENV) penetrates host cells. The virus is internalised by clathrin-mediated endocytosis once it has been bound. The E protein experiences conformational changes in the acidic environment of the cell, exposing the fusion loop and promoting membrane fusion, which permits viral RNA to enter the cytoplasm. In the interim between further optimisation and clinical research, membrane fusion inhibitors that target various viral protein areas have been created and are being assessed for possible therapeutic application^50^.

#### 5.2.2 Replication and transcription inhibitors

Balapiravir, which is generated from the nucleoside analogue R1479, has been shown in a randomized, double-blind, placebo-controlled experiment to be effective in treating dengue virus infection by selectively targeting the RNA-dependent RNA polymerase (RdRP) domain of the viral NS5 protein. When balapiravir was given to adult dengue patients within 48 hours of the onset of the disease, it did not considerably lower viremia, NS1 antigenemia, or fever clearance time in comparison to the placebo group. The trial found no significant differences in whole blood transcriptional patterns or plasma cytokine profiles between the treatment and placebo groups, despite the medication being well tolerated. The trial's overall failure to establish balapiravir as a viable treatment option for dengue virus infection highlights the difficulties in creating antiviral treatments that specifically target conserved viral proteins like NS5^51^.

**Figure 2:**
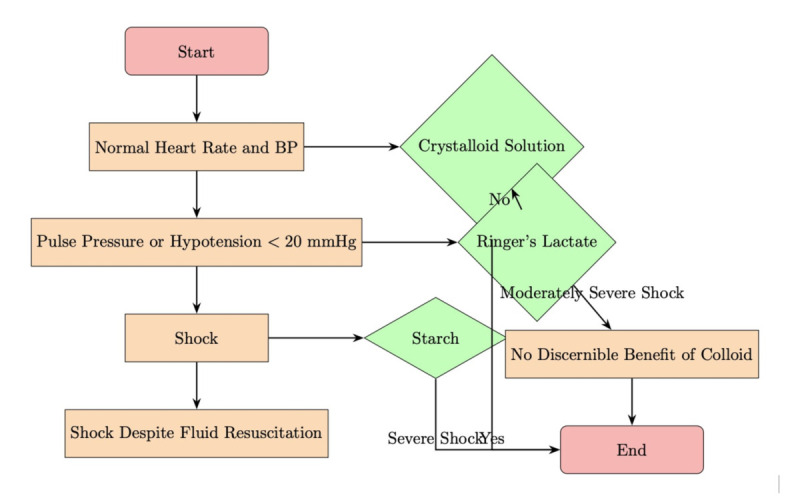
Fluid management for Dengue^47,48^

#### 5.2.3 Methyltransferase inhibitors

Because of its critical function in RNA synthesis and capping, which are necessary for viral stability and immune evasion, dengue virus NS5 is a prime target for treatments. Although specific inhibitors for the NS5 methyltransferase domain have been found, specificity and toxicity issues have made clinically meaningful capping inhibitors difficult to find. The review suggests creative enzyme engineering techniques to create therapeutic proteins that target NS5 to remedy this. Viral RNA may be more susceptible to host immune detection if engineered methyltransferases improve viral RNA binding and cause aberrant methylation. By lowering viral load and boosting immune response, these modified proteins may be administered using cutting-edge technologies like nanoparticles or chimeric proteins, potentially leading to better dengue treatment outcomes^52^.

#### 5.2.4 RNA-dependent RNA polymerase inhibitors

Ivermectin, a well-known antiparasitic drug, has shown promise against dengue virus in vitro by inhibiting host nuclear import proteins crucial for the nuclear localization of the dengue NS5 protein, which has RNA-dependent RNA polymerase (RdRp) function. A phase 2/3 randomized, double-blind, placebo-controlled trial investigated the efficacy of ivermectin at a once-daily dose of 400 μg/kg for 2–3 days in adult dengue patients^53^. The study found that while ivermectin treatment led to faster clearance of NS1 antigenemia, there was no significant difference in viremia, viral clearance, or any beneficial clinical outcomes such as fever, dengue hemorrhagic fever (DHF) incidence, hospitalization, pleural effusion, hemoconcentration, or fluid requirements. In order to assess ivermectin's potential as a dengue treatment, more study is required to comprehend its pharmacodynamics and mechanism of action with regard to NS1, as elevated NS1 levels are a risk factor for DHF.

#### 5.2.5 Nucleoside analog (NITD008)

Both in vitro and in vivo, NITD-008, a nucleoside inhibitor that targets RNA-dependent RNA polymerase, demonstrated a potent inhibitory impact against all four DENV serotypes. However, the acute renal toxicity seen in preclinical tests put a stop to its development^54^.

#### 5.2.6 Helicase inhibitors

The NS3 protein's C-terminal domain, which spans amino acids 180–618, performs the activities of a non-processive NTPase/helicase. This helicase is a member of the "SF2" helicase superfamily. For DENV, YFV, and JEV, helicase/NTPase activity and crystal structures have been described. Between subdomains 1 and 2, where ATP substrate binding predominates, is the location of the ATPase active site^55^.

#### 5.2.7 Protease inhibitors

Potential protease inhibitors for dengue virus NS2B/NS3 protease (PR) include diaryl (thio)ethers. With low micromolar IC50 values, benzothiazole derivatives selectively and noncompetitively suppress the dengue virus PR serotypes 2 and 3 in vitro and in cells. Specifically, these drugs target DENV replication while leaving HCV and HIV-1 unaffected. According to molecular docking, binding occurs at an allosteric location. Cell-based experiments demonstrate their potential to limit dengue virus multiplication at low or submicromolar concentrations by confirming their inhibitory ecacy^56^.

#### 5.2.8 NS4B inhibitor

As a direct-acting treatment for dengue virus, Janssen Pharmaceuticals' NS4B inhibitor JNJ-A07 has demonstrated encouraging preclinical results. JNJ-A07, which is derived from a ketoindole molecule, works by severing the link between the NS4B and NS3 proteins to prevent the development of a DENV replication complex. In vitro, it demonstrates antiviral efficacy against several clinical isolates including all four dengue serotypes. Even with a delayed start of therapy, it quickly lowers viremia and the amount of virus in organs in murine models and lowers pro-inflammatory cytokine level while raising survival rates^57^. However, more research is required to determine any potential negative consequences, such as decreased antibody production that could result in severe dengue in subsequent infections. Phase 2 clinical trials are now being conducted on JNJ-64281802, an analogue of JNJ-A07, for dengue prophylaxis in healthy individuals and therapeutic usage in patients with confirmed dengue fever. The purpose of these trials is to evaluate its safety and effectiveness in actual use.

#### 5.2.9 Drugs targeting host factors required by DENV to complete its life cycle

5.2.9.1 Chloroquine

The major aim of clinical studies for the widely used medication chloroquine (CQ), which is used to treat inflammatory illnesses such as lupus and rheumatoid arthritis, was the reduction of viral load. Research conducted in vitro indicates that CQ obstructs the pH-dependent phases of viral replication, meaning it prevents the growth of coronaviruses, retroviruses, and flaviviruses. CQ suppresses TNF-α and IL-6 production, which in turn prevents endosomal fusion and furin-dependent viral maturation during dengue virus (DENV) infection. But in a randomized, double-blind, placebo-controlled study, 307 adult Vietnamese patients infected with DENV were shown to have a longer viremia duration and no discernible decrease in the development of severe dengue hemorrhagic fever (DHF) in those treated with CQ. Additionally, there was a noticeable increase in vomiting that was linked to CQ treatment. A smaller, more recent trial on 37 dengue-positive patients revealed that although CQ treatment lessened the intensity of pain symptoms, it was unable to shorten the length of the illness or the intensity of the fever. These results raise questions regarding the possible negative effects of CQ and point to its limited effectiveness in treating dengue infection^58^.

5.2.9.2 Elgosiver

The alpha-glucosidase antagonist celgosivir protected mice against a deadly DENV challenge and showed abnormalities in the folding and ensnaring of viral NS1 in the endoplasmic reticulum. A phase 1b randomized placebo-controlled trial could not clearly demonstrate a benefit in lowering viremia or the course of the disease, despite the fact that DENV-infected mice treated with celgosivir showed reduced viremia and a mortality benefit. Further research on the pharmacokinetics and dose of the medication revealed that raising the dosage could enhance the medication's effectiveness^59^.

5.2.9.3. Statins

A class of medications known as statins prevents the synthesis of cholesterol by blocking the enzyme HMG-CoA reductase. Statins have anti-inflammatory characteristics in addition to their ability to decrease cholesterol. Since membrane lipids are essential to the Flavivirus life cycle, it was thought that statins would prevent the virus from replicating. This is corroborated by research showing statins have antiviral and anti-inflammatory qualities in a dengue-infected animal model. But a study evaluating lovastatin in DENV infection was unable to show any improvement in viremia or the duration of the illness^60^.

### 5.3 Alternative methods

#### 5.3.1 Revolutionary Methods for Aiming at the Vector

The introduction of genetically modified male mosquitoes, which sterilize the wild-type female population and lower egg production and the size of the following generation's population that could be available for possible dengue virus transmission, is one of the new vector-control strategies^61^. An alternate tactic is to introduce strains of the obligatory intracellular bacteria Wolbachia into A. aegypti during the embryonic stage. Remarkably, Aegypti with Wolbachia infection exhibits partial resistance to dengue virus infection and can infiltrate native populations of A.aegypti, indicating the potential for inducing pervasive biologic immunity against dengue viruses in A. aegypti populations^62-64^.

#### 5.3.2 Sofosbuvir

Due to shortcomings with current interventions such as the Dengvaxia vaccination, research into dengue fever treatments has increased. Nucleoside inhibitors that target the dengue virus polymerase are one line of inquiry; one such inhibitor is sofosbuvir, a prodrug known to be anti-HCV. Its active metabolite, GS-461203, has been found to exhibit strong viral suppression in both in vitro and in silico investigations, as well as a high binding affinity to the dengue viral polymerase's catalytic motif. The study also highlights the need for particular liver enzymes to activate sofosbuvir into its active form, highlighting potential complications in its therapeutic use against dengue. These results provide encouraging opportunities for the development of successful anti dengue treatments by highlighting the need for additional study into sofosbuvir's effectiveness and potential as a DENV polymerase inhibitor in human subjects^65^.

#### 5.3.3 Carica papaya

Plenty of companies are creating or formulating C. papaya leaf extract products that are sold in stores. Rochway, Herbal Papaya, SidoMuncul Herbal, and more companies prepare supplements. They are utilizing fermentation, micronization, and liquid extraction in the formulation of supplements^66.^ Numerous plant species may elevate platelet count. In several animal dengue fever models, C. papaya leaf extract has been shown to increase platelet count and shorten the clotting time in thrombocytopenic rats^67,68^. When fed orally to AG129 dengue-infected mice, freeze-dried C. papaya leaves also elevated plasma monocyte chemoattractant protein-1 (MCP-1) levels during the peak of viremia, revealing the potential immunomodulatory activity of this plant during DENV infection^69,70^.

#### 5.3.4 Azadirachta indica

A real-time polymerase chain reaction (RT-PCR) test using an aqueous extract of Azadirachta indica leaves at a maximum non-toxic concentration of 20–30 mg/mL revealed complete inhibition of viral replication, as did the absence of virus-specific 511 bp amplicon and DENV-related symptoms in DENV-infected suckling mice^71,72^.

#### 5.3.5 Vitamin E supplementation

Clinical investigations have demonstrated the benefits of vitamin E administration in addition to standard therapy, as it can reduce liver function abnormalities and promote platelet count recovery during dengue illness^73^.

### 5.4 Symptomatic management

It is advised to give large amounts of oral fluids and administer paracetamol as needed for antipyretic treatment during the febrile period. Avoid using any other nonsteroidal anti-inflammatory medications^74^. The patient can be maintained at home with daily full blood counts if there is a nearby medical facility available to them. Severe prostration, early bleeding symptoms, or excessive vomiting or diarrhoea that causes dehydration are all signs that the patient needs to be admitted to the hospital for close supervision^75^.

#### 5.4.1 Antiviral Research in cure of Dengue

Since there are currently no effective treatments for dengue, research into the development of antiviral medicines has accelerated in recent years. Small molecule inhibitors have traditionally targeted proteins such as NS3 protease, NS3 helicase, NS4B, and NS5. The NS3 and NS5 proteins are considered to be the most important targets for the development of antivirals due to their essential enzymatic roles in the process of viral replication. While NS5 possesses methyltransferase (MTase) and RNA-dependent RNA polymerase (RdRp) activities,NS3 has a variety of enzymatic activities, such as serine protease, nucleoside triphosphatase (NTPase), 5′-RNA triphosphatase, and helicase activities^76-78^.

#### 5.4.2 Corticosteroids

Corticosteroids have been debated for their potential role in dengue shock syndrome and the early administration to prevent progression to severe illness; however, there is insuffiecnt evidence for the support of this claim. Both natural immunological responses of phagocytes and a wide variety of immune responses mediated by T and B cells are inhibited by corticosteroids. High-dose corticosteroids are helpful in a variety of immunological abnormalities, including autoimmune illnesses like systemic lupus erythematosus. Corticosteroids used in the early acute phase of dengue infection did not affect shock, plasma leakage, or the recovery of platelet count in dengue patients, according to a double-blind, placebo-controlled trial. Neither the kinetics of dengue virological indicators nor the amounts of plasma cytokines were altered by it.

#### 5.4.3 Blood products

A common characteristic of Dengue Virus (DENV) infection is thrombocytopenia, which is defined as a platelet count below 150 × 10^9/l. Thrombocytopenia usually occurs between days 3 and 8 after symptom onset. As the hematocrit rises and platelet counts decrease, the disease is moving towards its critical phase. During defervescence (days 3–6), nadir platelet counts are attained, and then there is a gradual, spontaneous rebound. Mucosal or petechiae bleeding is common and typically goes away on its own without treatment. To reduce the risk of bleeding, severe thrombocytopenia (≤20 × 10^9/l) may require rigorous bed rest, avoidance of nonsteroidal anti-inflammatory medications, and intramuscular injections. Although preemptive platelet transfusion is frequently used in the treatment of sepsis, there is no proof of its effectiveness in dengue infections.Severe bleeding, frequently in the gastrointestinal or vaginal tract, is linked to metabolic acidosis, protracted shock, liver or renal failure, and certain drugs. Prompt transfusion of platelets, fresh frozen plasma, and packed red blood cells may be life-saving in such situations. However, because there are no long-term advantages and several serious concerns, including fluid overload and transfusion-related problems, prophylactic platelet transfusion is not advised.

#### 5.4.4 Alternative Measures for Treating Dengue Patients' Bleeding

Recombinant factor VIIa (rFVIIa) was shown to decrease bleeding temporarily in a research study including 25 children with dengue hemorrhagic fever (grade II–III) and active bleeding, but there was no overall effect. While there is some indication that intravenous anti-D globulin can improve platelet counts in dengue patients, there is not enough data to support the use of intravenous immunoglobulin (IVIg), interleukin-1 (IL-1), or tranexamic acid.

#### 5.4.5 Intravenous immunoglobulins

One recognised treatment Recombinant factor VIIa (rFVIIa is intravenous immunoglobulins (IVIG). Platelet-associated IgG (PAIgG) autoantibodies are responsible for thrombocytopenia in ITP. Platelets coated with PAIgG are rapidly cleared by means of Fcγ receptors that are found on mononuclear phagocytic cells. IVIG most likely occurs through ligation of inhibitory receptors or competitive inhibition of Fcγ receptors^79^. Thirty six dengue patients participated in a randomized, controlled study to assess IVIG treatment in relation to the amount and frequency used to treat ITP over the course of four days. The effectiveness of IVIG in increasing platelet recovery was not demonstrated by the trial^79^.

#### 5.4.6 Mast cell inhibitors

The pathophysiology of dengue-vascular leakage and hemorrhage has been linked to mast cell (MC) activation, according to recent research by St John et al.^48^. In a mouse model of DENV infection, it was demonstrated that activated MC releases a variety of proteases into the serum, most notably tryptase and chymase, which causes vascular integrity to be lost. The authors also demonstrated a correlation between serum chymase levels and dengue severity in people participating in a prospective research, which is in line with their observation in mice. Subsequently, the group demonstrated that, in spite of a little (but not statistically significant) rise in mouse viremia, MC-stabilizing drugs, such as cromolyn, montelukast, and ketotifen, decreased vascular leakage in the wild-type mice model of DENV assault^67^. All of these findings point to the possibility of using MCs as therapeutic targets to reduce DENV pathogenesis^47^.

## 6. Dengue Vaccine

The lack of specific antiviral treatment against dengue has placed a significant emphasis on preventative measures which include vector control and most importantly, vaccination. In combating and adequately controlling this disease, the development of vaccines has been an essential focus.

### 6.1 Advancements in the vaccine development

Considerable progress has been made in the development of vaccines against dengue in the last few decades. Most notably, the first licensed dengue vaccine known as Dengavaxia by San Pasteur’s became approved in various countries where dengue is an epidemic. Dengvaxia is a live-attenuated vaccine and is formulated to provide protection and immunity against all four serotypes of dengue^80^.

Additional advancements have led to the development of alternatives to Dengvaxia and aim to become established as safer and more effective contenders while simultaneously addressing concerns related to the safety profile of Dengvaxia. These advancements have been domadey utilizing various advanced approaches such as viral vectors, recombinant proteins and DNA vaccines.

### 6.2 Types of vaccines

#### 6.2.1 Live attenuated vaccines

Using weakened forms of viruses which are attenuated to reduce their virulence, live-attenuated vaccines can produce an immune response in the host. Sanofi Pasteur's Dengvaxia is an example of a live atta enuated vaccine and is approved to provide immunity against all four serotypes of dengue. Dengvaxia was developed by substituting the pRM/E RNAs of the yellow fever vaccine strain with equivalent sequences from the various serotypes. The vaccine has been licensed by several countries where dengue is endemic such as in Asia and Latin America^80,81^. Dengvaxia provides an immunity to the host which lasts for more than four years and clinical trials have revealed it to be more effective in individuals over the age of 9 ^82^.

#### 6.2.2 Recombinant protein vaccines

The production of recombinant protein vaccines ininvolveshe use of various genetic engineering techniques for the production of viral proteins, mainly the envelope proteins. These proteins are then modified to produce an immune response against dengue virus in the host. Several vaccine candidates have been developed utilizing this approach including a Tetravalent Dengue Vaccine (TAK-003). In comparison to the more widely used live-attenuated vaccines, recombinant protein vaccines are capable of inducing a more fitting immune response while simultaneously lowering the incidence of antibody-dependent enhancement^83,84^.

#### 6.2.3 Viral vector vaccines

To stimulate immune response, viral vector vaccines use genetically engineered viruses such as adenovirus or chimeric viruses to deliver the dengue virus into the body. Examples include NIH the live attenuated teta-valent vaccine (TV003/TV005) and IMCB’s chimeric dengue vaccine. Using a viral vector vaccine remains superior at inducing cellular immunity and has the potential to become better at causing robust humoral immune response^85^.

#### 6.2.4 DNA vaccine

Several dengue DNA vaccines are underway because they are stable and relatively easy to produce^86,87^.

### 6.3 Notable Examples

#### 6.3.1 Dengue Virus (DENV) Vaccines Dengvaxia

This technology facilitated the development of four chimeric YF-DEN viruses, which were utilized in the development of a tetravalent DENV vaccine. The vaccine is based on the 17D strain of yellow fever virus, where the pre-membrane and envelope proteins of yellow fever virus have been substituted by the corresponding genes from each of the four DENV serotypes. These serotypes were originally obtained from DENV isolates collected in Thailand and Indonesia between 1978 and 1988^19,20^. After one injection of the vaccine, either at a high or low dose, in cynomolgus macaques, it produced seroconversion and significant neutralizing antibody responses against all four DENV serotypes and limited viremias in comparison to the parental DENV strains. It's interesting to note that 92% of the immunized monkeys were shielded from a challenge with wild-type DENV 1-4 by challenge experiments^88^.

#### 6.3.2 TV003/TV005

Given the significance of untranslated regions (UTRs) in the replication of the DENV genome, the first attenuation method concentrated on removing 30 consecutive nucleotides (172–143) from the 3 ´-UTR of DENV-4 (rDEN4∆30) in the TL2 stem-loop^89^. For DENV-1, rDEN1∆30—a mutant lacking the same homologous genomic region—was also created. When confronted with wild types of DENV-1 and DENV-4, both mutants showed an attenuated phenotype as evidenced by their decreased infectivity and their ability to elicit robust neutralizing antibody responses in rhesus macaques, which coincided with the protection^90^.

Continued efforts towards development of tetravalent DENV vaccine included vrrating an attenuated DENV-2 component. This was achieved by utilizing rDEN4∆30 backbone to create two attenuated chimeric viruses: one with the membrane and envelope genes (rDEN2/4 ∆30 (ME)), and another with the capsid, membrane, and envelope genes (rDENV2/4 ∆30 (CME)) similarly substituted^91^.

#### 6.3.3 TAK-003 (DENVax)

When scientists at Mahidol University in Bangkok, Thailand, identified a DENV-2 strain (DENV-2 16681) from a patient’s serum, they began developing the DENVax vaccine in the late 1980s. The DENV-2 PDK-53-V strain was obtained by attenuating the DENV-2 16681 strain in primary dog kidney cells (PDK cells) through 53 serial passages. This strain differs from the parental DENV-2 PDK-53 strain in that it has an additional non-synonymous mutation in the NS3 gene, while attenuation-related mutations are present in the 5 ´UTR and NS1 gene. The DENV-2 PDK53-V strain was also the foundation for the development of the DENVax vaccines, exhibiting decreased neurovirulence in nursing mice and decreased replication rates in C6/36 cells^92^.

### 6.4 Challenges in the development of vaccine

Despite the recent advancements and progress, the development of a vaccine against dengue faces many challenges. A significant challenge is the requirement of induction of simultaneous immunity against all four serotypes of dengue. Vaccine development is further complicated by the phenomenon of antibody-dependent enhancement, in which prior infection with one serotype may amplify and exacerbate disease upon subsequent infection with another serotype of dengue. Furthermore, safety concerns have been raised against Dengvaxia because of reports of severe dengue infection in vaccinated individuals without any prior history of exposure and infection. These reports have impacted the trust of the public in the dengue vaccination programs and have prompted regular scrutiny. Maintaining efficacy and addressing safety concerns at the same time is a critical challenge for the developers.

Additional challenges in vaccine development are posed by the complex epidemiology of the disease, with varying prevalence of serotypes in different populations and variable transmission dynamics. This requires tailored vaccination programs for different populations and equitable access to vaccines in endemic areas, particularly low- and middle-income countries where prevalence of dengue is the highest. This represents additional challenges to the infrastructure of the healthcare system, distribution and economic affordability.

### 6.5 Future of vaccine development

Despite the multiple challenges, ongoing research has offered promising results to the development of safe and effective vaccine development coupled with a better understanding of the epidemiology and immunology of the disease that provide help in overcoming the different hurdles. Addressing these challenges requires collaboration and efforts of researchers, policymakers and healthcare professionals. Additionally, innovative strategies should be employed to minimize antibody-dependent enhancement, to increase immunogenicity and to provide cross-protective efficacy.

## 7. Conclusion

In conclusion, this comprehensive review provides a deeper understanding of the complexities and multifaceted nature of this infectious disease through an exploration of its pathogenesis, clinical features and emerging insights. Amidst the challenges posed by the wide spectrum of clinical presentations and complications, ongoing research efforts have yielded valuable insights into the immunological mechanisms underlying the viral infection, role of host genetics in disease susceptibility and the development of therapeutic interventions. Additionally, the development of dengue vaccine offers hope for reducing the burden of dengue fever worldwide. A review on dengue is essential for clinicians and healthcare professionals to stay updated and informed about the pathogenesis, treatment protocols and prevention strategies i.e., vaccines.
